# Expanding the phenotype in argininosuccinic aciduria: need for new therapies

**DOI:** 10.1007/s10545-017-0022-x

**Published:** 2017-03-01

**Authors:** Julien Baruteau, Elisabeth Jameson, Andrew A. Morris, Anupam Chakrapani, Saikat Santra, Suresh Vijay, Huriye Kocadag, Clare E. Beesley, Stephanie Grunewald, Elaine Murphy, Maureen Cleary, Helen Mundy, Lara Abulhoul, Alexander Broomfield, Robin Lachmann, Yusof Rahman, Peter H. Robinson, Lesley MacPherson, Katharine Foster, W. Kling Chong, Deborah A. Ridout, Kirsten McKay Bounford, Simon N. Waddington, Philippa B. Mills, Paul Gissen, James E. Davison

**Affiliations:** 1 Gene Transfer Technology Group, Institute for Women’s Health, University College London, London, UK; 2 Metabolic Medicine Department, Great Ormond Street Hospital for Children NHS Foundation Trust, Great Ormond Street, WC1N 3JH London, UK; 3 Genetics and Genomic Medicine Programme, Great Ormond Street Institute of Child Health, University College London, London, UK; 4 Metabolic Medicine Department, Royal Manchester Children Hospital NHS Foundation Trust, Manchester, UK; 5 Metabolic Medicine Department, Birmingham Children’s Hospital NHS Foundation Trust, Birmingham, UK; 6 North East Thames Regional Genetic Services, Great Ormond Street Hospital NHS Foundation Trust, London, UK; 7 Charles Dent Metabolic Unit, National Hospital for Neurology and Neurosurgery, London, UK; 8 Metabolic Medicine Department, Evelina Children’s Hospital, London, UK; 9 Metabolic Medicine Department, St Thomas Hospital, London, UK; 10 Paediatric Metabolic Medicine, Royal Hospital for Sick Children, Glasgow, UK; 11 Neuroradiology Department, Birmingham Children’s Hospital NHS Foundation Trust, Birmingham, UK; 12 Neuroradiology Department, Great Ormond Street Hospital NHS Foundation Trust, London, UK; 13 Population, Policy and Practice Programme, UCL Institute of Child Health, London, UK; 14 West Midlands Regional Genetic Laboratory, Birmingham Women’s Hospital, Birmingham, UK; 15 Wits/SAMRC Antiviral Gene Therapy Research Unit, Faculty of Health Sciences, University of the Witwatersrand, Johannesburg, South Africa; 16 MRC Laboratory for Molecular Cell Biology, University College London, London, UK

## Abstract

**Objectives:**

This UK-wide study defines the natural history of argininosuccinic aciduria and compares long-term neurological outcomes in patients presenting clinically or treated prospectively from birth with ammonia-lowering drugs.

**Methods:**

Retrospective analysis of medical records prior to March 2013, then prospective analysis until December 2015. Blinded review of brain MRIs. *ASL* genotyping.

**Results:**

Fifty-six patients were defined as early-onset (*n* = 23) if symptomatic < 28 days of age, late-onset (*n* = 23) if symptomatic later, or selectively screened perinatally due to a familial proband (*n* = 10). The median follow-up was 12.4 years (range 0–53). Long-term outcomes in all groups showed a similar neurological phenotype including developmental delay (48/52), epilepsy (24/52), ataxia (9/52), myopathy-like symptoms (6/52) and abnormal neuroimaging (12/21). Neuroimaging findings included parenchymal infarcts (4/21), focal white matter hyperintensity (4/21), cortical or cerebral atrophy (4/21), nodular heterotopia (2/21) and reduced creatine levels in white matter (4/4). 4/21 adult patients went to mainstream school without the need of additional educational support and 1/21 lives independently. Early-onset patients had more severe involvement of visceral organs including liver, kidney and gut. All early-onset and half of late-onset patients presented with hyperammonaemia. Screened patients had normal ammonia at birth and received treatment preventing severe hyperammonaemia. *ASL* was sequenced (*n* = 19) and 20 mutations were found. Plasma argininosuccinate was higher in early-onset compared to late-onset patients.

**Conclusions:**

Our study further defines the natural history of argininosuccinic aciduria and genotype–phenotype correlations. The neurological phenotype does not correlate with the severity of hyperammonaemia and plasma argininosuccinic acid levels. The disturbance in nitric oxide synthesis may be a contributor to the neurological disease. Clinical trials providing nitric oxide to the brain merit consideration.

**Electronic supplementary material:**

The online version of this article (doi:10.1007/s10545-017-0022-x) contains supplementary material, which is available to authorized users.

## Introduction

In the central nervous system, nitric oxide (NO) is involved in crucial processes including neurotransmission (Garthwaite 2008), neuronal differentiation (Peunova and Enikolopov 1995) and migration (Nott et al. 2013). Argininosuccinate lyase (ASL) cleaves argininosuccinate into arginine and fumarate as part of the NO-citrulline cycle that regulates NO production in multiple tissues (e-Figure 1) (Nagamani et al. 2012a). ASL deficiency causes argininosuccinic aciduria (ASA; OMIM 207900), the only inherited condition proven to cause systemic NO deficiency (Erez et al. 2011). ASL is also required for the liver-based urea cycle, which detoxifies ammonia produced by amino acid catabolism. ASA is the second most common urea cycle defect (UCD) with an incidence of 1:70,000 live-births (Nagamani et al. 2012a) and presents clinically either as an early neonatal-onset (<28 days of age) hyperammonaemic coma, or a later-onset hyperammonaemic crisis (Nagamani et al. 2012a). A chronic phenotype with neurocognitive, gastrointestinal and liver symptoms without severe hyperammonaemia is also recognised (Nagamani et al. 2012a). Conventional treatment aims to decrease ammonia by use of a protein-restricted diet and ammonia scavenger drugs (sodium benzoate and phenylbutyrate) and to correct arginine deficiency by L-arginine supplementation (Haberle et al. 2012).

The phenotype in ASA differs from other UCD by the higher incidence of neurocognitive symptoms, liver fibrosis, renal impairment and systemic hypertension (Nagamani et al. 2012a; Kolker et al. 2015). These symptoms are observed in patients with early- or late-onset forms and in those without documented episodes of hyperammonaemia (Marble et al. 2008). Among UCD, ASA patients have the lowest frequency of hyperammonaemic crises (23%) but the second highest frequency of cognitive impairment (65–74%) after arginase deficiency (Ruegger et al. 2014; Waisbren et al. 2016). This paradox raises questions about the role of hyperammonaemia in causing the neurological problems. Newborns screened and treated prospectively from birth have been reported to have a better neurological outcome (Widhalm et al. 1992; Ficicioglu et al. 2009; Mercimek-Mahmutoglu et al. 2010). As conventional treatment decreases ammonia levels, it was suggested that neurological complications were caused by unrecognised hyperammonaemic episodes (Widhalm et al. 1992). However, newborn screening programmes can capture a wide phenotypic spectrum, including patients who would remain asymptomatic without treatment. Some of these screened patients had high residual ASL activity (Ficicioglu et al. 2009), suggesting that the prospectively treated cohort might have had an increased number of mild cases, introducing a bias into the comparison.

We describe a United Kingom (UK) wide cohort of ASA patients expanding the disease natural history, reporting long-term neurological outcomes with a focus on neuroimaging and genotype–phenotype correlations. The outcomes in patients treated prospectively (10/56) were compared with those who presented with symptoms before diagnosis (46/56).

## Material and methods

### Patients

Anonymised data were collected prospectively from March 2013 and retrospectively before, from five tertiary metabolic centres in the UK: Birmingham Children’s Hospital, Birmingham; Guy’s and St Thomas’ Hospital, London; Great Ormond Street Hospital for Children, London; the National Hospital for Neurology and Neurosurgery, London; the Royal Manchester Children Hospital, Manchester. Molecular analysis of patients was approved by the National Research Ethics Service Committee London-Bloomsbury (13/LO/0168). Patients included had plasma argininosuccinic acid levels > 5 μmol/L, and/or pathogenic mutations in *ASL*. Patients were considered lost to follow-up if no clinical assessment was performed during the last 3 years at the relevant metabolic centre. The database was closed on 31st December 2015.

Neurological outcome was assessed with physical neurological examination regarding developmental impairment, epilepsy, ataxia, myopathy-like symptoms and brain MRI features and was performed as follows: if neuropsychological assessment was unavailable, cognitive impairment was determined by clinical judgement of the metabolic specialist or neuropaediatrician or by the need for additional support in school or subsequently at the workplace. Epilepsy was defined as the occurrence of two or more seizures without accompanying hyperammonaemia. MR spectroscopy was performed as described previously (Davison et al. 2011). Indication for neuroimaging was an unexplained and/or severe neurological disease. Brain MRIs were analysed by two neuroradiologists blinded to the report of each other. Magnetic resonance spectroscopy (MRS) studies were performed concurrently with clinically indicated MRI scans at 1.5 T. Comparison was made with MRS metabolite data from a standard cohort of children with normal appearing MRI as described previously. Liver involvement was considered using the following parameters: hepatomegaly, increased levels of transaminases (alanine aminotransferase ALT > 50 IU/L). Nephromegaly was defined as renal length on ultrasound imaging above the 95th centile for the age and sex. Biochemical data were assessed using the mean of at least the last ten results available during compensated metabolic state. Plasma ammonia levels were considered elevated if >100 μmol/L before 28 days of life or >45 μmol/L subsequently. Hypokalaemia was defined as a plasma potassium level lower than 3.5 mmol/L and judged as “transient” if observed in a single sample, or “persistent” if measured in ≥ 2 samples separated by ≥ 1 month. Plasma arginine and argininosuccinic acid reflect the last ten measurements performed during follow-up in a compensated metabolic state on the patient’s standard treatment. For analysis, patients were divided into three groups: (i) early-onset form (hyperammonaemic symptoms started on/before 28 days of life), (ii) late-onset form (presentation after 28 days of life), (iii) perinatally screened patients diagnosed after a family proband and treated prospectively from birth. For the last group, the status (early- or late-onset) of the familial proband was investigated but missing data prevented inclusion of these index cases into the study.

### *ASL* sequencing

The 16 coding exons of *ASL* (NM_001024943.1; Ensembl ENST00000395332) and the intron-exon boundaries were PCR-amplified. Sequencing performed with the Big Dye Terminator Cycle Sequencing System version 1.1 (Applied Biosystems/ThermoFisher Scientific) was run on an ABI PRISM 3730 DNA Analyzer (Applied Biosystems/ThermoFisher Scientific) (e-Methods).

### Statistical analysis

Statistical analyses used Fisher’s exact test for investigating the association between categorical data and the patient groups (www.vassarstats.net). Continuous variables between groups were compared using the Student’s *t* test or one-way ANOVA with Bonferroni correction for pairwise comparison (*p* values detailed in e-Table 1) (GraphPad Prism 5.0, San Diego, CA, USA). *p* values ≤ 0.05 were considered statistically significant. Kaplan-Meier survival curves were compared with the log-rank test. Patients 10, 18 and 52, who died during the first month of life, and patient 53, for whom very limited clinical information was available, were excluded from long-term analysis. For patients lost during follow-up (*n* = 6), the assessments at their last follow-up visits were used for analysis.

## Results

### Patients

Fifty-six patients were classified as early-onset (*n* = 23/56), late-onset (*n* = 23/56) or screened patients (*n* = 10/56). Ethnic origins were White British (*n* = 24; 44%), Pakistani (*n* = 16; 29%), Chinese (*n* = 6; 11%), Indian (*n* = 5; 9%), Bangladeshi (*n* = 3; 5%), other White European (*n* = 1; 2%) and missing data (*n* = 1; 2%) (e-Table 3). Screened patients, diagnosed either antenatally or neonatally, had an affected familial proband with early-onset (*n* = 5), late-onset (*n* = 3) or unknown (*n* = 2) phenotype (e-Table 3). One pair of siblings with early-onset form (patients 15 and 16) was included in the study. Mean follow-up was not significantly different between the early-onset (EO) or late-onset (LO) groups compared to the screened group (SCR) (*p* = 0.19 and *p* = 0.19 respectively) (Table 1).

**Table 1:** Epidemiological and clinical data for the three analysed cohorts: early-onset, late-onset and screened patients

			Early onset	Late onset	Screened	Total
Epidemiology		Number (adult)	23 (3 = 10%)	23 (16 = 70%)	10 (1 = 10%)	56 (20 = 36%)
		Sex (M/F)	12/11	11/12	8/2	31/25
		Consanguinity	12/23 (52%)	2/23 (9%)	5/8 (62%)	21/52 (40%)
		Patients still living	16/23 (73%)	21/23 (91%)	9/10 (90%)	46/56 (82%)
At diagnosis		Age	4 days (2–8)	2.75 years (0.25–12)	2 antenatally 8 neonatally	2 years (0–12)
		Ammonia (RI < 100 μmol/L if <28 days of life; <50 μmol/L if >28 days of life)	861 ± 120	212 ± 67	84 ± 18	530 ± 85
Follow up		Mean follow-up (years)	11 (1.9–25.7)	15.1 (1–53)	15.6 (8–18.2)	12.4 (0–53)
		Patients lost	2/23 (9%)	4/23 (18%)	2/10 (20%)	8/56 (14%)
		Age (years)	11 (1.9–25.7)	23 (16.7–57)	15.6 (8–18.2)	15.6 (1.9–57)
Phenotype	Neurology	Developmental delay	18/21 (86%)	23/23 (100%)	7/8 (88%)	48/52 (92%)
		Age when first reported (years)	2 (0.1–4)	2.5 (1–6)	3.1 (2–4)	2 (0.1–6)
		Epilepsy	8/21 (38%)	11/23 (48%)	3/8 (38%)	22/52 (42%)
		Age when first reported (years)	9 (1.5–13)	2 (0.7–11)	8.5 (8–9)	5.5 (0.7–13)
		Ataxia	3/21 (14%)	6/23 (26%)	0/8 (0%)	9/52 (17%)
		Myopathic features	4/21 (19%)	3/23 (13%)	0/8 (0%)	7/52 (13%)
		Abnormal brain MRI	5/9 (56%)	5/10 (50%)	2/4 (50%)	12/23 (52%)
	Liver	Hepatomegaly	17/21 (81%)	3/23 (13%)	5/8 (62%)	25/51 (49%)
		Age when first reported (years)	2.5 (0–12)	13.0	07.5 (0.9–11)	2.5 (0–12)
		Raised ALT	18/21 (86%)	4/23 (17%)	6/8 (75%)	28/51 (55%)
		Age when first reported (years)	0.15 (0–6)	23 (1–53)	7 (3–7)	1 (0–53)
		ALT (RI 20–50 IU/L)	238 ± 77	81 ± 24	181 ± 50	169 ± 37
	Kidney appearance (ultrasound)	Enlargement (>95th centile)	8/18 (44%)	2/9 (22%)	2/5 (40%)	12/32 (38%)
		Poor corticomedullar differentiation	2/7 (29%)	0/3 (0%)	1/2 (50%)	3/12 (25%)
	Miscellaneous	Hypokalaemia: total	16/23 (70%)	4/23 (17%)	6/10 (60%)	26/56 (46%)
		Persistent	3/23 (13%)	3/23 (13%)	1/10 (10%)	7/56 (12%)
		Intermittent	13/23 (56%)	1/23 (4%)	5/10 (50%)	19/56 (34%)
		Arterial hypertension	1	0	0	1
		Age when first reported (years)	11	/	/	11
		Trichorrhexis nodosa	0/23 (0%)	5/23 (22%)	0/10 (0%)	5/56 (9%)
		Age when first reported (years)	/	7.3 ± 2.2	/	
		Severe diarrhoea	10/21 (48%)	3/20 (15%)	4/10 (40%)	17/51 (33%)
Biology	Plasma arginine (RI 30-126 μmol/L)		126 ± 19	102 ± 12	134 ± 15	116 ± 9
	Plasma argininosuccinic acid (RI <5 μmol/L)		512 ± 92	234 ± 64	238 ± 206	356 ± 62
Therapeutics	Protein restricted diet	Frequency	20/20 (100%)	16/20 (80%)	6/7 (86%)	42/47 (89%)
		Daily protein allowance (g/kg/day)	1.2 ± 0.14	1.2 ± 0.1	1.4 ± 0.08	1.2 ± 0.07
	L-arginine supplementation	Frequency	20/20 (100%)	20/20 (100%)	7/7 (100%)	47/47 (100%)
		L-arginine (mg/kg/day)	239 ± 28	155 ± 25	251 ± 45	201 ± 20
	Na benzoate supplementation	Frequency	17/19 (89%)	2/22 (9%)	6/7 (72%)	25/48 (52%)
		Na benzoate (mg/kg/day)	215 ± 18	134 ± 33	167 ± 17	191 ± 15
	Na phenylbutyrate supplementation	Frequency	7/19 (37%)	2/22 (9%)	1/7 (14%)	10/48 (21%)
		Na phenylbutyrate (mg/kg/day)	200 ± 50	57 ± 12	NA	143 ± 31

Age at diagnosis, currently, at first occurrence of symptom and duration of follow-up are presented as median ± range. Other figures show mean ± standard error. Hypokalaemia-total includes patients with intermittent and persistent hypokalaemia. Follow-up is considered until December 2015. Plasma arginine and argininosuccinic acid concentrations reflect the last ten measurements performed during follow-up when patients were in a compensated metabolic state on their standard treatment

*ALT* alanine aminotransferase, *NA* not available, *RI* range interval

### Neurological phenotype

The frequency and median age of onset of the neurological features were not significantly different between the groups (e-Table 1).

Developmental impairment was reported in 48/52 patients (92%) and was the most common symptom. The median age at diagnosis was 2 years (range 0.1–6 years) (Table 1) and when observed, developmental impairment was present before the age of 6 years in all but two patients. Only four patients were reported with normal neurocognitive function: three early-onset patients aged <6 months (patient 22), 23 months (patient 15) and 11 years old at last assessment (patient 11) and one patient screened at birth (patient 47; sibling to a late-onset proband, aged 8 years old at last assessment) (e-Table 2). Developmental impairment was mild or moderate affecting predominantly speech and learning ability. Detailed information about schooling was available in 35 patients (e-Table 2). Only 6/35 patients (17%) attended mainstream school without the need for additional educational support (patients 9, 11, 35, 36, 47 and 48 with last assessment at 25, 11, 22, 20, 8 and 16 years old respectively). Most patients (20/35; 57%) required speech and language therapy. The neuropsychological assessments identified behavioural difficulties with auto- or hetero-aggression (*n* = 3) and learning disabilities in logic and reasoning. Twenty-one adults (EO *n* = 3; LO *n* = 17; SCR *n* = 1) with a median age of 22.3 years (range 18–57 years) were assessed for socioeconomic status. Five (25%) had semi-skilled employment. Independent living was reported in 1/12 (8%), and long-term relationships in 3/12 (25%). Patients not living independently were accommodated in the parental (9/11; 82%) or care (2/11; 18%) homes.

Epilepsy was observed in 22/52 patients (42%) with no significant difference of the median age of onset between groups (Table 1). Various seizure types were reported including generalised, partial and complex, febrile and afebrile seizures. Tonic-clonic seizures were most frequent (*n* = 16), followed by absence seizures (*n* = 5), myoclonic jerks (*n* = 4), atonic seizures (*n* = 2) and occasionally status epilepticus (*n* = 1) (e-Table 2). Electroencephalogram was performed in three non-epileptic patients and showed an abnormal pattern in two patients. 17/22 patients (77%) were treated with an average of 1.5 antiepileptic drugs (range 0–4).

Cerebellar dysfunction was detected in early-onset and late-onset patients (*n* = 3 and *n* = 6 respectively) with the incidence of 9/52 (17%) (Table 1). Ataxia was first noticed at a median age of 8.5 years (range 1–12) with two main age groups at first observation, early around the age of 1 year (*n* = 2) or later as teenagers (*n* = 7). Three patients had dyskinesia and tremor and one had nystagmus (e-Table 2). The mild inconvenience caused did not require any specific medical or surgical treatment.

Episodes of myopathy-like symptoms, reported in 7/52 patients (13%) (Table 1), included global hypotonia with a hypomimic facial expression, and unexplained recurrent episodes of general weakness persisting several days before spontaneous recovery. One patient (patient 28, currently aged 15.9 years) was reported with fatigable ptosis from 12 years old onwards. Four patients were investigated with electromyogram, which were always normal, including a Tensilon test in one patient. One patient (patient 33 currently aged 26 years) presented with an electrophysiologically confirmed episode of Guillain-Barré syndrome at 8 years old (e-Table 2).

**Fig. 1:**
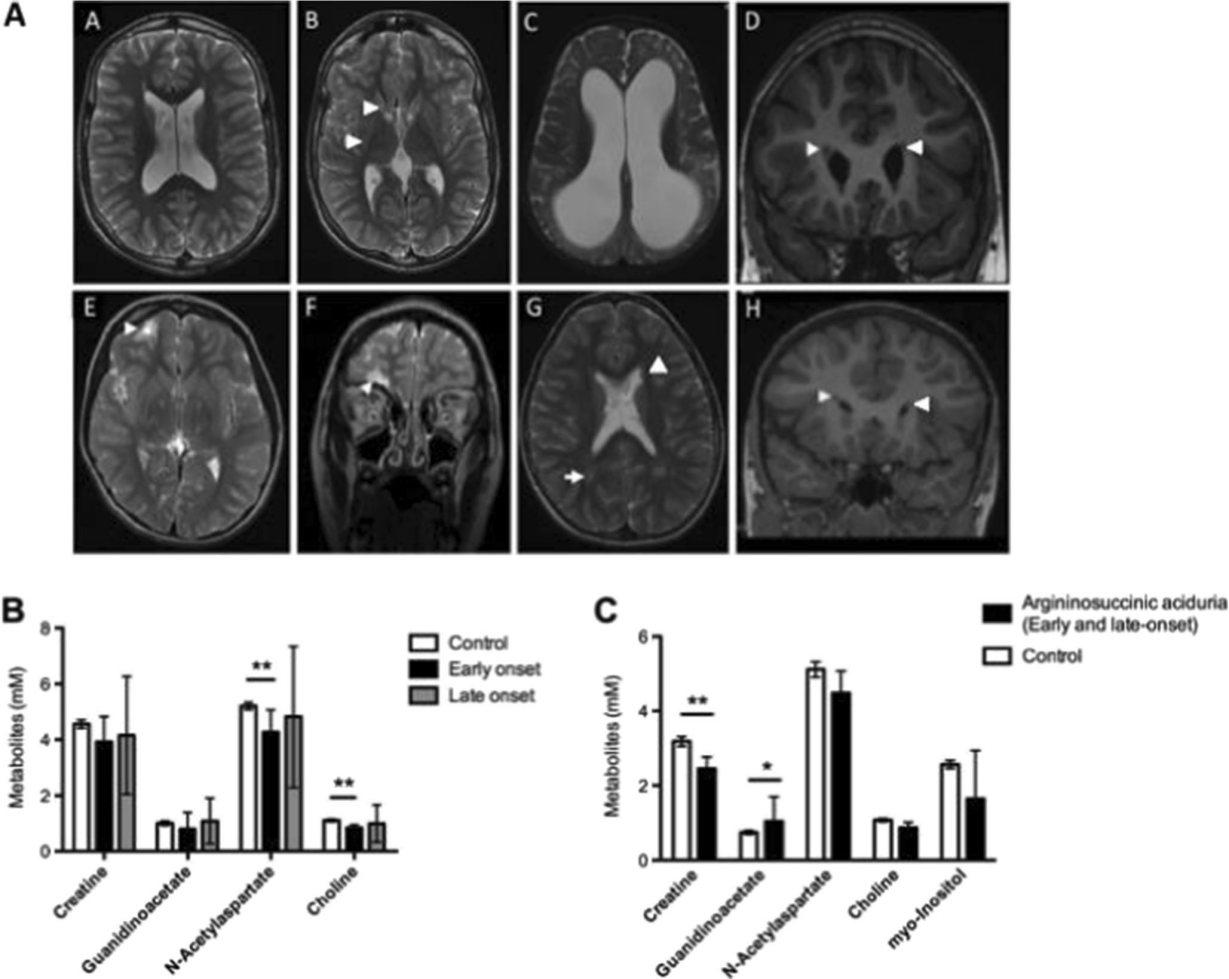
Neuroimaging. **A**: Morphological brain MRI features. *A*, *B*: T2-weighted axial images showing brain matter volume loss and mild ex vacuo dilatation of ventricles (*A*) and high signal in bilateral caudate heads and posterior putamina (*arrows*). *C*: T2-weighted axial images with severe diffuse cerebral atrophy and ventricular dilatation. *D*, *H*: T1-weighted coronal image with right periventricular heterotopia (*arrowheads*). *E*, *F*: T2-weighted axial (*E*) and coronal (*F*) images with evidence of right inferior frontal lobe infarct (*arrow*). *G*: T2-weighted axial image with bilateral high signal of the peritrigonal white matter (*arrow*). **B** ^1^ *H* MR spectroscopy features in basal ganglia. Assessment in early-onset (*n* = 5), late-onset (*n* = 3) and control (*n* = 63) patients analysed using a paired t test. **c** ^1^ *H* MR spectroscopy features in white matter. Patients affected by argininosuccinic aciduria (*n* = 4) and controls (*n* = 53) analysed with one way ANOVA. *Graphs* represent mean ± 95% confidence interval. * *p* < 0.05; ** *p* < 0.01

MRI brain was performed as part of the clinical work-up in 21 patients with unexplained or severe neurological features. The average age at the time of MRI was 12 years (range 0–23). Twelve scans were reported as abnormal (52%) (Table 1). Neuroimaging performed during follow-up showed small parenchymal infarcts (*n* = 4), foci of white matter hyperintensity on T2-weighted sequences (*n* = 4), nodular heterotopia (*n* = 2), cortical atrophy (*n* = 2), cerebellar atrophy (*n* = 2), perirolandic gliosis (*n* = 1), thalamic atrophy (*n* = 1), hyperintensity of caudate head and posterior putamen (*n* = 1) or isosignal between pallidi and putamen (*n* = 1) (Fig. 1A and e-Table 2). Spectroscopy of basal ganglia (*n* = 8; 3 early-onset, 5 late-onset) indicated a significant decrease of N-acetylaspartate and choline in early-onset patients compared to controls (*p* = 0.001 and *p* = 0.008, respectively). Creatine and guanidinoacetate levels in the basal ganglia did not differ significantly between controls, early- or late-onset groups (Fig. 1B). Spectroscopy of the white matter (*n* = 4; 3 early-onset, 1 late-onset) showed a significant decrease in creatine levels (*p* = 0.003) and an increase of guanidinoacetate (*p* = 0.01) (Fig. 1C) in patients compared to controls.

### Systemic phenotype

**Fig. 2:**
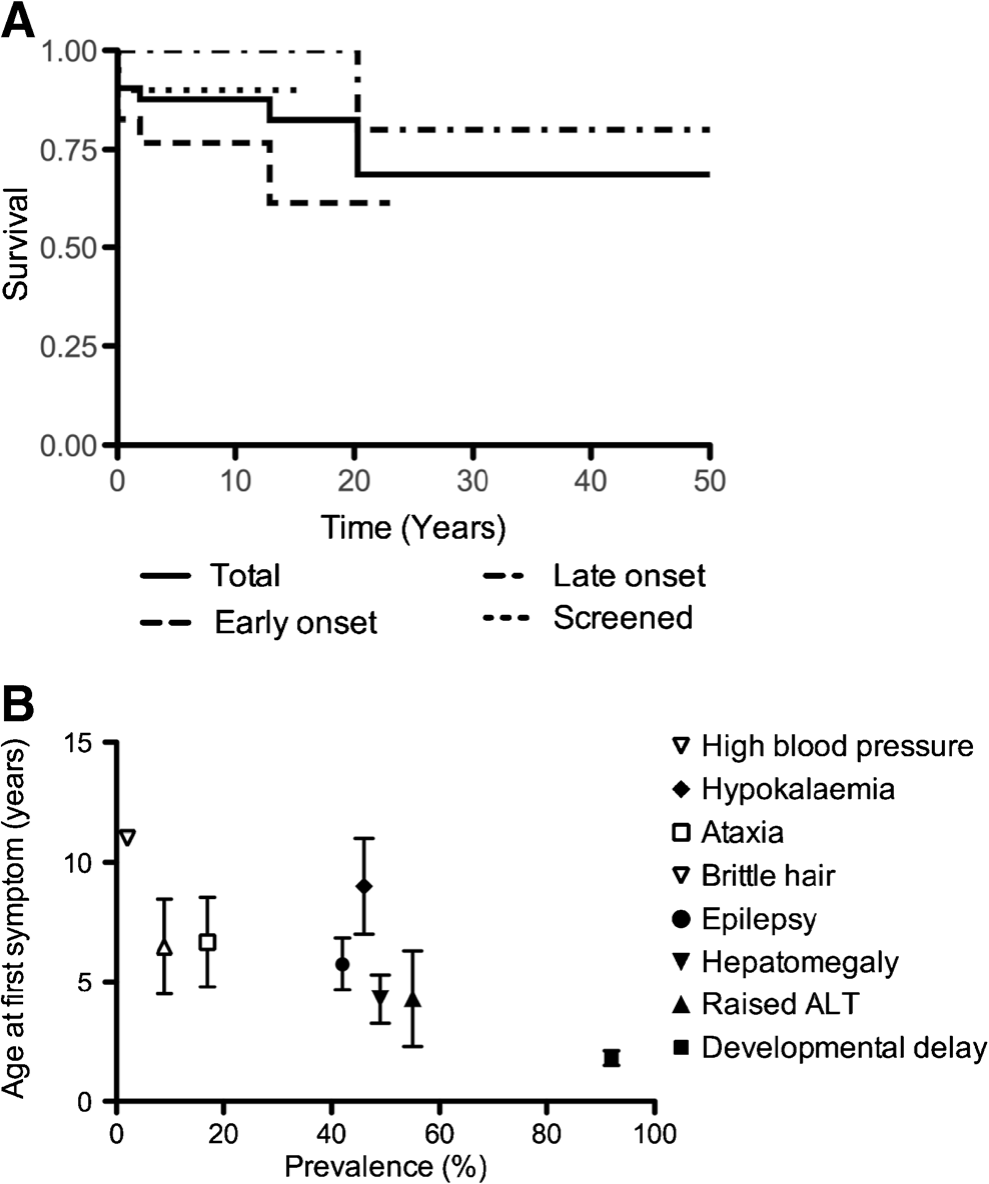
Natural history of argininosuccinic aciduria. **A** Kaplan-Meier survival curves for all (*solid line*), early-onset (*dashed line*), late-onset (*dashed dotted line*) and screened (*dotted line*) patients. **B** Natural history of the systemic phenotype of argininosuccinic aciduria. Mean ± standard error of age of onset of each symptom from data of the whole cohort when information available: developmental delay (*n* = 7), abnormal LFTs (*n* = 8), hepatomegaly (*n* = 18), epilepsy (*n* = 15), brittle hair (*n* = 4), ataxia (*n* = 6), hypokalaemia (*n* = 2), high blood pressure (*n* = 1). Symptom frequency in the total population of patients studied is presented in brackets. ALT: plasma alanine aminotransferase activity. It was assumed that patients had normal blood pressure if hypertension was not specifically mentioned in medical records

47/56 patients (84%) were alive at the time of assessment with no significant difference between groups, with a median follow-up of 12.4 years (range 0–53) (Fig. 2A). Cause and age of death were hyperammonaemic decompensation at presentation (*n* = 2; patients aged day 3 and 4 of life), sepsis (*n* = 3; patients aged 7 days, 11 years and 20 years), extradural hematoma (*n* = 1; patient aged 2 years), hepatocellular carcinoma (*n* = 1; patient aged 4.5 years), acute pancreatitis (*n* = 1; patient aged 12 years) and a possible arrhythmia (*n* = 1; patient aged 52 years).

Natural history data included the age of onset of organ involvement or symptoms (Fig. 2B). Among neurological symptoms, developmental delay was the first observed usually during the second or third year of life followed by epilepsy and ataxia.

The commonest hepatic involvement was a persistent rise in plasma alanine transaminase activity, usually accompanied by hepatomegaly. These were significantly more frequent in early-onset and screened patients than in late-onset patients (*p* < 0.00001 and *p* < 0.005, respectively; e-Table 1). In screened patients, the likelihood of hepatic abnormalities depended on the age of onset of the disease in the familial proband: 4/4 of screened patients with an early-onset familial history had hepatic abnormalities compared to 1/3 with a familial late-onset phenotype (Table 1).

There was no evidence of differences between groups for nephromegaly and poor corticomedullary differentiation assessed by ultrasound (Table 1). Transient or persistent hypokalaemia occurred more frequently in early-onset versus late-onset patients (*p* < 0.03) (Table 1). Acute metabolic decompensation, gastroenteritis and acute diarrhoea were significantly associated with transient hypokalaemia (*p* < 0.005).

Trichorrhexis nodosa was observed only in late-onset patients before diagnosis and normalised with treatment (Table 1).

Chronic profuse diarrhoea was observed in 17 patients (33%; including early-onset *n* = 10, late-onset *n* = 3, screened patients *n* = 4; Table 1). This symptom was refractory to symptomatic and immunosuppressive treatments and caused nutritional difficulties in several early-onset patients. Two patients had colonoscopies performed at 5 years of age and repeated at ages 7 and 10. Intestinal biopsies showed non-specific mild inflammation. Chronic pancreatitis was observed in one early-onset patient.

Refractory arterial hypertension was diagnosed in one early-onset patient (patient 6) at the age of 9 years and was sub-optimally controlled despite three antihypertensive medications. This patient died at 12 years old from acute pancreatitis. One late-onset patient developed atrial flutter at 60 years.

### Ammonaemia, ASA levels and therapies

None of the patients in the screened group suffered severe or prolonged hyperammonaemia. Three of these patients had an initial ammonia level >100 μmol/L (133, 134 and 190 μmol/L), which normalised in less than 24 hours. All early-onset patients had hyperammonaemia at diagnosis with values significantly higher than in the late-onset and screened groups (*p* < 0.001; e-Table 1). Only 50% of the late-onset patients were hyperammonaemic at diagnosis.

A protein-restricted diet was used in 89% of patients (100% of the early-onset group and 80% of the late-onset group; Table 1). All patients were treated with L-arginine with no significant difference in the dose between groups (Table 1). Ammonia scavenger drugs (sodium benzoate and phenylbutyrate) were prescribed significantly more often in the early-onset and screened groups than in the late-onset group (*p* < 0.001 and *p* < 0.01 respectively) and at higher doses in the early-onset group (*p* = 0.03) (e-Table 1).

Plasma argininosuccinate levels were higher in early-onset (512 ± 92 μmol/L) compared to late-onset (234 ± 64 μmol/L) (*p* = 0.03) (e-Table 1).

### Genotype–phenotype correlation

The genotype was available for 19 patients (Table 2). Twenty mutations (including eight novel) were identified: 11 were missense, five splice site, two nonsense mutations and two deletions. A sequence alignment of *ASL* showed that all missense mutations affect amino acid residues that are highly conserved across species. The deletions included a 13 base pair deletion (c.1045_1057del, p.(Val349Cysfs*72)) and one large deletion of approximately 2 kb which included exons 15 and 16 (c.1143+117_*1353del). Homozygous mutations observed with early onset disease included c.437G>A p.(Arg146Gln), c.749T>A p.(Met250Lys), c.1045_1057del p.(Val349Cysfs*3), c.1143+117_*1353del and c.1153C>T p.(Arg385Cys). Homozygous mutations observed with late-onset disease included c.35G>A p.(Arg12Gln), c.377G>A p.(Arg126Gln) and c.1138A>G p.(Lys380Glu). The c.1045_1057del deletion is predicted to cause a frameshift and introduction of a premature stop codon and the c.1143+117_*1353del deletion is predicted to cause the loss of exons 15 and 16, and both were associated with early-onset phenotype. Patients homozygous for c.1143+117_*1353del were younger brothers of a proband who was not genotyped but presented with the early onset phenotype (Table 2).

**Table 2:** Genotype–phenotype correlation of *hASL*

Patient number	Allele 1	Allele 2	Presumed effect on protein		Severity in this study	Reported severity in the literature
1	c.35G>A	c.35G>A	p.(Arg12Gln)	p.(Arg12Gln)	Late onset	Unknown (*n* = 1) (Balmer et al. 2014)
2	c.348+1G>A	c.532G>A	Splicing effect	p.(Val178Met)	Late onset	New genotype
3	c.349-1G>A	c.532G>A	Splicing effect	p.(Val178Met)	Early onset	New genotype
4, 5	c.377G>A	c.377G>A	p.(Arg126G1n)	p.(Arg126G1n)	Late onset	New genotype
6	c.437G>A	c.437G>A	p.(Arg146G1n)	p.(Arg146G1n)	Early onset	New genotype
7	c.437G>A	c.446+1G>A	p.(Arg146G1n)	Splicing effect	Late onset	New genotype
8	c.544C>T	c.772G>A	p.(Arg182^*^)	p.(Glu258Lys)	Late onset	New genotype
9	c.719-2A>G	c.857A>G	Splicing effect	p.(Gln286Arg)	Early onset	New genotype
10	c.721G>A	c.918+5G>A	p.(Glu241Lys)	Splicing effect	Early onset	New genotype
11, 12	c.749T>A	c.749T>A	p.(Met250Lys)	p.(Met250Lys)	Early onset	New genotype
13	c.1045_1057del	c.1045_1057del	p.(Val349Cysfs*72)	p.(Val349Cysfs*72)	Early onset	New genotype
14	c.1138A>G	c.1138A>G	p.(Lys380Glu)	p.(Lys380Glu)	Late onset	Unknown (*n* = 1) (Balmer et al. 2014)
15, 16^a^	c.1143+117_^*^1353del	c.1143+117_^*^1353del	Loss of exons 15 and 16	Loss of exons 15 and 16	Early onset	New genotype
17, 18	c.1153C>T	c.1153C>T	p.(Arg385Cys)	p.(Arg385Cys)	Early onset	Prenatal diagnosis (*n* = 3, Keskinen et al. 2008; Balmer et al. 2014), early onset (*n* = 4, this study, Kleijer et al. 2002; Keskinen et al. 2008), late onset (*n* = 7, Kleijer et al. 2002; Keskinen et al. 2008), unknown (*n* = 3, Balmer et al. 2014)
19	c.1284G>A	c.1366C>T	p.(Trp428^*^)	p.(Arg456Trp)	Late onset	New genotype

New genotype refers to combination of mutations not described before. Presumed protein effect is mentioned within brackets for novel mutations. For screened patients, the severity of the phenotype is deducted from the symptomatic familial proband

^a^Patients 15 and 16 are siblings

## Discussion

This study describes three groups of ASA patients (early-onset, late-onset and perinatally screened after a familial proband) with prolonged follow-up periods and compares the long-term outcome with regard to the time at initiation of treatment. In contrast to previously reported patients diagnosed by newborn screening, this study describes screened patients, who had a familial index case with a known phenotype.

### Neurological outcome

The most common complications of ASA were neurological. Comparison between groups demonstrates a homogeneous long-term neurological outcome, with no significant difference in frequency, severity and age of onset for all neurological features assessed. Previously unreported neuroimaging findings such as focal infarcts or heterotopia might be related to impaired NO-dependent neuronal migration or microcirculation.

The early-onset group had higher ammonia levels compared to the late-onset group, as evidenced by differences in the ammonia levels at diagnosis, and need for ammonia scavenger medications and protein restriction. The screened patients also needed more treatment to control their ammonia levels than patients in the late-onset group because most of them (5/8) had siblings with early-onset disease. Plasma ASA levels were higher in early-onset compared to late-onset and screened patients. However, these differences did not affect the neurological phenotype. These observations show that hyperammonaemia and ASA levels are not the dominant factors causing the long-term neurological phenotype in ASA. Previous publications have suggested that neonatal screening and early treatment may prevent or ameliorate the neurological disease (Widhalm et al. 1992; Ficicioglu et al. 2009). However, an extended Austrian cohort of neonatally screened patients, initially reported with normal neurocognitive outcome at a mean age of 6 years (Widhalm et al. 1992), showed that 35% of patients (6/17; median age 13 years) had an IQ of less than 80 (Mercimek-Mahmutoglu et al. 2010). As the neurological disease is progressive, duration of the follow-up is essential to determine the outcome objectively. At the end of the first year, the frequency of developmental impairment is similar to other UCD (64%) attributable to sequelae of neonatal hyperammonaemia in early-onset patients (Burgard et al. 2016). This frequency increases to 65–100% with time (this study; Keskinen et al. 2008; Tuchman et al. 2008; Ah Mew et al. 2013; Ruegger et al. 2014).

Aggressive behaviour and psychiatric problems such as psychosis and paranoid ideation were previously reported in ASA (von Wendt et al. 1982; Odent et al. 1989; Lagas and Ruokonen 1991; Sijens et al. 2006), although these features were not observed in this cohort.

### Systemic phenotype

Our study found a wide range of systemic complications. All of them were more frequent in the early-onset group, apart from trichorrhexis nodosa, although blood pressure was not systematically investigated. Chronic diarrhoea, not reported previously, was a major problem in many patients with endoscopy showing mild inflammation. This has been observed in an enterocyte-specific conditional knockout mouse model, in which a loss of ASL was associated with necrotizing enterocolitis (Premkumar et al. 2014). A similar pattern of systemic complications was found in the screened group, suggesting that prospective treatment has no preventative effect.

### Genotype–phenotype correlation

Mutation analysis of our cohort identified genotype/phenotype correlation for some of the mutations in agreement with the literature.

The frequently occurring mutation c.35G>A p.(Arg12Gln) was associated with the late-onset phenotype in one patient as previously suggested for homozygous and compound heterozygous patients (Sampaleanu et al. 2002; Mercimek-Mahmutoglu et al. 2010; Balmer et al. 2014). It has been reported that the arginine 12 residue on the N-terminal loop close to the catalytic site of ASL might influence the binding/exit of the substrate without affecting the catalytic site explaining the milder phenotype (Sampaleanu et al. 2002).

The novel homozygous mutation c.749T>A p.(Met250Lys) was observed in two unrelated patients with early-onset phenotype. This mutation involves changes in the protein sequence close to two other amino acid modifications also associated with early-onset phenotype p.(Glu241Lys) and p.(Trp245fs) (Balmer et al. 2014).

The homozygous mutation c.1153C>T p.(Arg385Cys) has been associated previously with both early-onset (*n* = 4) (Kleijer et al. 2002; Keskinen et al. 2008) or late-onset phenotypes (*n* = 7) (Kleijer et al. 2002; Keskinen et al. 2008). However, all patients with late-onset phenotype were diagnosed before 20 months of life. p.(Arg385Cys) has been reported as a founder mutation in the Finnish population (Keskinen et al. 2008) and is associated with very low ASL activity affecting an amino acid near the catalytic site (Hu et al. 2015).

### Pathophysiology of ASA

Various pathophysiological mechanisms have been proposed to account for the long-term complications of ASA. Argininosuccinic acid may be toxic to the brain, either directly or via the formation of guanidino compounds. Raised guanidinoacetate was reported on brain spectroscopy of ASA patients in the grey (3.63 ± 0.6 mmol/L) and the white matter (3.52 ± 0.09 mmol/L) (Sijens et al. 2006; van Spronsen et al. 2006) and may be explained by L-arginine supplementation (Sijens et al. 2006). In our study, levels of guanidinoacetate were similar to controls in basal ganglia but slightly elevated in white matter (1.05 ± 0.41 mmol/L). Patients with guanidinoacetate methyltransferase (GAMT) deficiency have much higher guanidinoacetate concentrations in brain (3.4-3.6 mmol/L) (Stockler et al. 1994) and CSF (11–12 μmol/L) (Stockler-Ipsiroglu et al. 2014). There is also some evidence of raised guanidinoacetate in patients with hyperargininaemia due to Arginase deficiency, with variable CSF guanidinoacetate concentrations (up to 0.127 μmol/L versus controls 0.049 μmol/L) (Deignan et al. 2010), although the spectral peak of guanidinoacetate at 3.8 ppm was not seen in a cohort of adult patients with hyperargininaemia (Carvalho et al. 2014), while in a 3 year old patient a prominent peak at 3.8 ppm was ascribed to arginine, which in vitro has resonances at 3.75 and 3.23 ppm (Wishart et al. 2007) and may have masked any detectable guanidinoacetate. Guanidinosuccinic acid can be neurotoxic (D’Hooge et al. 1992) and activates N-methyl-D-aspartate receptors (Aoyagi et al. 2001). However, this hypothesis is not supported by the observation of the early-onset group, which has higher levels of argininosuccinic acid but neurological outcomes similar to the other groups.

In humans, ASL is crucial for the synthesis of L-arginine, which becomes an essential amino acid in ASA. Arginine deprivation, associated with altered NO-mediated immune responses, can lead to site-specific neuronal loss in animal models of neurodegenerative diseases (Kan et al. 2015). Arginine is a precursor for the synthesis of creatine and agmatine (e-Figure 1). Brain spectroscopy showed creatine deficiency (this study; Sijens et al. 2006; van Spronsen et al. 2006; Boenzi et al. 2012). However, the role of secondary creatine deficiency in cerebral dysfunction has not been convincingly demonstrated (Boenzi et al. 2012). Agmatine is involved in learning (Leitch et al. 2011), neuroprotection (Molderings and Haenisch 2012) and anticonvulsant effect (Demehri et al. 2003). Thus, secondary agmatine deficiency could explain some of the neurological symptoms.

Finally, several symptoms may be caused by impaired NO synthesis. Using an *Asl* ^*Neo/Neo*^ mouse model, Erez et al. (2011) showed that defective ASL is responsible for the loss of the catalytic function of the enzyme and affects the structure of a multi-protein complex incorporating NOS. This disrupts the NOS-dependent NO synthesis and leads to systemic NO deficiency. Hypoargininaemia can lead to uncoupling of NOS, decreased NO production and increased generation of free radicals that damage tissues (e-Figure 1) (Nagamani et al. 2012b). Reactive oxygen species interfere with NO production and regulation of the microcirculation (Shu et al. 2015). In addition decreased NO levels might affect protein S-nitrosylation (Jaffrey et al. 2001), which in turn regulates histone methylation and gene expression (Nott and Riccio 2009). In the brain, NO plays a key-role as a signalling molecule (Riccio 2010) involved in neurotransmission (Garthwaite 2008), regulation of neuronal differentiation (Peunova and Enikolopov 1995; Lameu et al. 2012) and migration (Bredt and Snyder 1994; Nott et al. 2013). In human, NO therapy was reported to have mild neurocognitive benefit (Nagamani et al. 2012b). In this study, NO deficiency might account for the neuropathology underlying the neuroimaging findings such as local parenchymal infarcts or nodular heterotopia due to impaired microcirculation and abnormal neuronal migration during development respectively. Besides neurological implications, NO is involved in various physiological processes such as vasodilatation (Cosby et al. 2003), liver fibrosis (Diesen and Kuo 2011), muscle strength and performance (De Palma et al. 2014), kidney filtration rate (Satriano et al. 2008) and gut physiology (Vallance and Charles 1998; Premkumar et al. 2014; Bogdan 2015). Therefore, NO deficiency might be involved at least partially in various symptoms highlighted in this study including chronic hepatitis, myopathy-like phenotype, chronic diarrhoea and systemic hypertension.

### Optimising therapeutics in ASA

This study demonstrates persisting neurological and systemic disease not obviously related to hyperammonaemia in ASA patients on conventional treatment. Although some organs (liver, kidney, gut) are more frequently affected in early-onset patients, who have higher ammonia and ASA levels, this is strikingly not the case for the brain. Our observation of parenchymal infarcts, nodular heterotopia and the report of mild neurological improvement after NO therapy (Nagamani et al. 2012b) support the role of NO deficiency in the pathophysiology of the brain disease in ASA. Currently correction of NO deficiency is not considered in the conventional treatment of ASA. Liver transplantation (Marble et al. 2008; Newnham et al. 2008) cures the urea cycle but would not be expected to correct the systemic NO-arginine cycle defect. Similarly, successful liver-targeted gene therapy in *Asl* ^*Neo/Neo*^ mouse did not correct extra-hepatic features such as defective NO-mediated vascular relaxation (Nagamani et al. 2012b). Future therapeutic approaches in ASA might consider targeting the NO deficiency, which could include the use of an enriched nitrate diet, nitrate therapy (Nagamani et al. 2012b; Erez 2013) or multiorgan-targeted gene replacement.

## Electronic supplementary material

Below is the link to the electronic supplementary material.

e-Figure 1 (DOCX 194 kb)

e-Table 1 (DOCX 145 kb)

e-Table 2 (DOCX 291 kb)

e-Table 3 (DOCX 269 kb)

ESM 1 (DOCX 50 kb)

## Abbreviations

ALT: Alanine aminotransferase
ASA: Argininosuccinic aciduria
ASL: Argininosuccinate lyase
CSF: Cerebro spinal fluid
NO: Nitric oxide
NOS: Nitric oxide synthase
UCD: Urea cycle defects
