# Supplementary material for: Expanding the phenotype in argininosuccinic aciduria: need for new therapies
Source: J Inherit Metab Dis. 2017 Mar 1;40(3):357–68. doi: 10.1007/s10545-017-0022-x (PMC5393288; doi:10.1007/s10545-017-0022-x)
Supplement: Supplementary file 1 — (DOCX 194 kb) [file 10545_2017_22_MOESM1_ESM.docx]

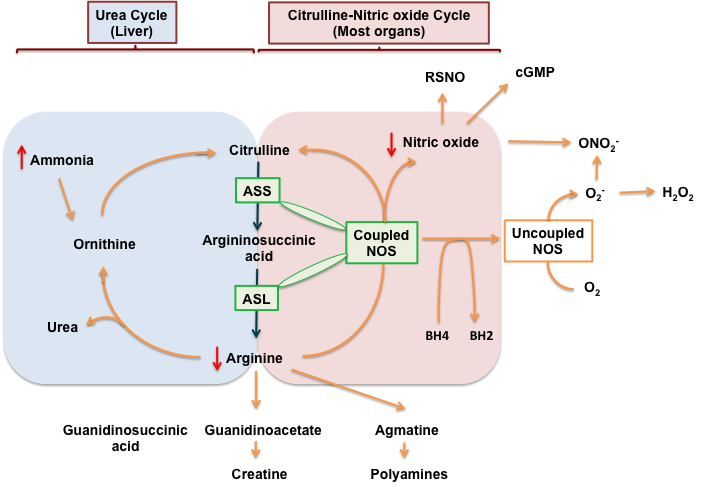


**e-Figure 1.** **Metabolic pathways affected by argininosuccinate lyase deficiency.** Argininosuccinate lyase (ASL) cleaves argininosuccinate into arginine and fumarate as part of the urea cycle in the liver and the citrulline-nitric oxide cycle in most of organs. ASL is part of a multiprotein complex including argininosuccinate synthase (ASS) and nitric oxide synthase (NOS). ASL deficiency leads to reduced arginine, increased ammonia and reduced nitric oxide levels. Nitric oxide acts on the nitric oxide-cyclic guanosine monophosphate signalling pathway to mediate its effects in a wide range of tissues and generates nitrosothiols (RSNO) through protein nitrosylation. Nitric oxide acts on the nitric oxide-cyclic guanosine monophosphate signalling pathway to mediate its effects in a wide range of tissues and generates nitrosothiols (RSNO) through protein nitrosylation. Low arginine or tetrahydrobiopterin (BH_4_) cause uncoupling of the NOS dimer generating superoxide. Superoxide forms other reactive oxygen species like hydrogen peroxide (H_2_O_2_) or peroxynitrite (ONO_2_^-^) involved in free radical damage and acting as signaling molecules. Arginine is a precursor for various pathways i.e creatine, agmatine and polyamine synthesis. Argininosuccinic acid could have a toxic effect by itself or through the formation of the toxic guanidinosuccinic acid. ASS: Argininosuccinate synthase; ASL: Argininosuccinate lyase; BH2: dihydrobiopterin; BH4: tetrahydrobiopterin; cGMP: cyclic guanosine monophosphate; H_2_O_2_ hydrogen peroxide; NOS: Nitric oxide synthase; O_2_^-^ Superoxide; ONO_2_^-^ peroxynitrite; RSNO: nitrosothiols.
