# Supplementary material for: Expanding the phenotype in argininosuccinic aciduria: need for new therapies
Source: J Inherit Metab Dis. 2017 Mar 1;40(3):357–68. doi: 10.1007/s10545-017-0022-x (PMC5393288; doi:10.1007/s10545-017-0022-x)
Supplement: Supplementary file 2 — (DOCX 145 kb) [file 10545_2017_22_MOESM2_ESM.docx]

**e-Table 1. Pair comparison for statistical tests**. The direction of the pair comparison is mentioned in brackets (EO for Early-onset). CI: confidence interval; ns: not significant.
