# Supplementary material for: Expanding the phenotype in argininosuccinic aciduria: need for new therapies
Source: J Inherit Metab Dis. 2017 Mar 1;40(3):357–68. doi: 10.1007/s10545-017-0022-x (PMC5393288; doi:10.1007/s10545-017-0022-x)
Supplement: Supplementary file 3 — (DOCX 291 kb) [file 10545_2017_22_MOESM3_ESM.docx]

**e-Table 2.** **Detailed neurological phenotype of patients in this study.** * Adult patient at end of follow-up in December 2015. For screened patients, the phenotype of the disease affected the familial proband is specified: (E) Early-onset, (L) Late-onset. A: Atonic; Ab: Absence; <DA/CA>: Developmental age/Current age; IQ: Intellectual quotient; M: Myoclonus; N: No; NA: Not available; NP: Not performed; Sz: seizure; TC: Tonic-clonic; TR: tendon reflexes Y: Yes; ?: Unknown.
