# Supplementary material for: Expanding the phenotype in argininosuccinic aciduria: need for new therapies
Source: J Inherit Metab Dis. 2017 Mar 1;40(3):357–68. doi: 10.1007/s10545-017-0022-x (PMC5393288; doi:10.1007/s10545-017-0022-x)
Supplement: Supplementary file 4 — (DOCX 269 kb) [file 10545_2017_22_MOESM4_ESM.docx]

**e-Table 3. Detailed general phenotype of the patients in this study.** M: Male; F: Female; NP: Not performed; Y: Yes; N: No; NA: Not available; cDNA: coding DNA. It was assumed that patients had normal blood pressure if hypertension was not specifically mentioned in medical records.
