# Supplementary material for: Expanding the phenotype in argininosuccinic aciduria: need for new therapies
Source: J Inherit Metab Dis. 2017 Mar 1;40(3):357–68. doi: 10.1007/s10545-017-0022-x (PMC5393288; doi:10.1007/s10545-017-0022-x)
Supplement: Supplementary file 5 — (DOCX 50 kb) [file 10545_2017_22_MOESM5_ESM.docx]

**e-Methods**

***ASL* sequencing**

Genomic DNA was extracted from venous leucocytes. Details of PCR primers (tailed) and conditions used for PCR amplification are available upon request. The prevalence of all variants identified was assessed in the Ensembl (http://ensembl.org) and ExAC databases (<http://exac.broadinstitute.org>), and their presence or absence in the Human Gene Mutation Database was examined (last accessed 11/12/2015; <http://www.hgmd.cf.ac.uk>). All protein alignments were performed using ClustalW2 software (http://www.ebi.ac.uk/Tools/msa/clustalw2/).
